# Long-Term Bond Strength of Two Benzalkonium Chloride-Modified Adhesive Systems to Eroded Dentin

**DOI:** 10.1155/2017/1207208

**Published:** 2017-08-10

**Authors:** Simon Flury, Adrian Lussi, Anne Peutzfeldt

**Affiliations:** Department of Preventive, Restorative, and Pediatric Dentistry, School of Dental Medicine, University of Bern, Freiburgstrasse 7, 3010 Bern, Switzerland

## Abstract

This study investigated the effect of benzalkonium chloride (BAC) modification of two adhesive systems on long-term bond strength to normal and artificially eroded dentin. A total of 128 extracted human molars were sectioned and the buccal and oral surfaces of each molar were ground until the dentin. One half was left untreated (normal dentin) while the other half underwent artificial erosion. Resin composite was bonded to the buccal or oral surface following treatment with Adper Scotchbond 1XT or OptiBond FL without or with 1% BAC incorporation. Shear bond strength (SBS) was measured after 24 h (100% humidity, 37°C) or 1 year (tap water, 37°C). SBS results were statistically analyzed (*α* = 0.05). SBS was significantly lower to artificially eroded dentin than to normal dentin (*p* < 0.001). Storage for 1 year had no effect on SBS to normal dentin but led to a significant decrease in SBS to artificially eroded dentin (*p* < 0.001). BAC incorporation decreased the 24 h SBS to normal dentin (*p* = 0.018), increased the 24 h SBS to eroded dentin (*p* = 0.001), and had no effect on the 1-year SBS for either substrate. Consequently, BAC incorporation did not improve bond durability.

## 1. Introduction

Studies have shown that the adhesive bond to dentin deteriorates over time [1–4], which may jeopardize the long-term durability of resin composite restorations. The main factor leading to reduction of the adhesive bond over time is hydrolysis of resin and collagen in the hybrid layer [2, 5]. The hydrolytic stability of adhesive systems differs between the various classes available: adhesive systems that include application of a separate adhesive resin layer (i.e., three-step etch-and-rinse and two-step self-etch adhesive systems) have proved more stable than adhesive systems that do not comprise such a layer (i.e., two-step etch-and-rinse and one-step self-etch adhesive systems) [2]. Being more hydrophilic, the latter adhesive systems act as semipermeable membranes, attract water, and thus degrade faster. Another factor leading to degradation of resin-dentin bonds is collagenolytic activity by matrix metalloproteinases (MMPs) in dentin. MMPs are endogenous enzymes that are released and activated when exposed to an acidic environment such as the one created by etching with phosphoric acid and/or application of acidic primers or adhesive resins [5–9]. MMPs, along with cysteine cathepsins that are capable of activating MMPs and of cleaving type I collagen, have been shown to be responsible for the hydrolytic degradation of the collagen matrix within the hybrid layer [5, 10]. Adhesive retention to dentin relies on infiltration of resin into the mineralized dentin. This infiltration requires removal of minerals by phosphoric acid or acidic monomers. The minerals are replaced by the water used to rinse off the phosphoric acid (etch-and-rinse adhesive systems) or by the water used as a solvent in the primers and/or adhesive resins. During application of the adhesive resin, solvated monomers are intended to replace the water and penetrate into and around collagen fibrils to result in hybridization [11]. Unfortunately, adhesive resins are not able to replace all water, and the bottom portion of the hybrid layer contains collagen fibrils that are only partially protected by resin [12, 13]. The water remnants and incomplete resin impregnation render the collagen fibrils and the hydrophilic resins vulnerable to hydrolytic degradation, the destruction of collagen fibrils being caused mainly by activation of the collagen-bound MMPs [3, 9, 11] and that of the resins by degradation of the ester bonds in the adhesive polymer. The gradual destruction of the hybrid layer is inevitably accompanied by a gradual loss of bond strength.

In an effort to retard endogenous enzymatic degradation of the resin-dentin bonds, numerous studies have explored the capacity of various protease inhibitors such as chlorhexidine and quaternary ammonium compounds for their ability to inhibit MMP activity. These inhibitors, either incorporated in the phosphoric acid used prior to application of etch-and-rinse adhesive systems or applied as a separate step after phosphoric acid etching, have been shown to inhibit MMPs and cysteine cathepsins, to prevent collagen degradation and preserve the integrity of hybrid layer collagen matrix [6, 14–19], and to reduce the time-dependent deterioration of the resin-dentin bond [17, 20–25]. Chlorhexidine and the quaternary ammonium compound benzalkonium chloride (BAC) have also been incorporated into adhesive primers [16, 26] or into the adhesive component itself [27, 28], thus avoiding an extra step in the application procedure and possibly prolonging their presence in the hybrid layer. When incorporated into primers/adhesives, both protease inhibitors were still capable of reducing collagen degradation within the hybrid layer and retarding bond deterioration, especially in relatively hydrophilic primers/adhesives [27] and provided that the concentration was sufficiently high [26]. Incorporation of 1% BAC even led to a higher immediate bond strength than was obtained with the control adhesive [28].

Bond strength to eroded dentin has been found not only to be lower than the bond strength to normal, sound dentin but also to be more adversely affected by aging [29]. Eroded teeth displaying exposed dentin very often need treatment with sealants or resin composite in order to prevent further loss of tooth substance [30–33]. Any improvement in bond strength to eroded dentin and in bond durability would therefore be of great clinical interest. Consequently, this study aimed to investigate the effect of incorporation of BAC on long-term bond strength of a two-step etch-and-rinse and a three-step etch-and-rinse adhesive system to normal or artificially eroded dentin.

The null hypotheses were that neither (1) BAC incorporation nor (2) type of dentin (i.e., normal or eroded) or (3) duration of storage (i.e., 24 hours or one year) would have any influence on bond strength for either adhesive system.

## 2. Materials and Methods

### 2.1. Preparation of Dentin Specimens

A total of 256 dentin specimens were prepared from 128 extracted human permanent molars without restorations or caries (*n* = 16 dentin specimens/group, 16 groups: two adhesive systems without/with BAC incorporation on normal or artificially eroded dentin stored for either 24 h or 1 year). Before extraction, patients had been informed about the use of the molars for research purposes and verbal consent had been obtained. After extraction, the molars were pooled. The local ethics committee categorizes pooled teeth as an “irreversibly anonymized biobank” and thus no previous ethical approval was needed. The molars were cleaned under tap water with a scaler to remove any debris and soft tissue and stored in 2% chloramine solution in the refrigerator (4°C) until needed.

For preparation of dentin specimens, the molars were apically shortened with a water-cooled diamond saw (IsoMet Low Speed Saw, Buehler; Lake Bluff, IL, USA) and then sectioned along the mesiodistal axis (IsoMet Low Speed Saw, Buehler) and finally wet-ground from the buccal and oral surfaces to obtain flat dentin surfaces. Grinding was performed with grit #220 followed by grit #500 silicon carbide (SiC) abrasive papers on a Struers LaboPol-21 grinding machine (Struers; Ballerup, Denmark). The oral and buccal halves of the molars were embedded in cylindrical stainless steel molds with self-curing acrylic resin (Paladur, Heraeus Kulzer GmbH, Hanau, Germany). Consequently, two dentin specimens per molar were obtained, and after removal of the steel molds, all dentin specimens were kept in a humid chamber (100% humidity) in the refrigerator (4°C). Before adhesive treatment and preparation of shear bond strength (SBS) specimens, one dentin specimen (i.e., one half) of each molar underwent artificial erosion.

### 2.2. Artificial Erosion of Dentin Specimens

The 128 dentin specimens that underwent artificial erosion were subjected to a cyclic de- and remineralization procedure over 7 days with 42 de- and remineralization cycles in analogy to a previous study [29] (6 cycles per 24 h, 4 h per cycle with 5 min demineralization, 3.5 h remineralization (remaining 25 min: rinsing with deionized water between each demineralization/remineralization)). The cyclic de- and remineralization procedure was performed in a custom-made pH-cycling machine and the composition of the de- and remineralization solutions [34] is listed in Table 1. The pH of the de- and remineralization solutions was checked daily. After the cyclic de- and remineralization procedure, all artificially eroded dentin specimens were kept in a humid chamber (100% humidity) in the refrigerator (4°C).

### 2.3. Preparation of SBS Specimens

One hour before adhesive treatment, the dentin specimens were retrieved from the refrigerator and kept in tap water at room temperature. The dentin surfaces of the artificially eroded halves of each molar were left untreated whereas the dentin surfaces of the other halves were wet-ground for 5 s with grit #500 SiC abrasive papers (Struers) to obtain a standardized smear layer, with the abrasive papers being changed after grinding of 8 dentin specimens. Subsequently, each dentin specimen was air-dried and the bonding area defined and isolated by use of self-adhesive tape with a perforation (diameter ~2 mm). The bonding area was then treated as listed in Table 2. For Adper Scotchbond 1XT and OptiBond FL with BAC, 60 mg benzalkonium chloride BioXtra (Sigma-Aldrich, St. Louis, MO, USA; CAS number: 63449-41-2, lot number: BCBP1900V) was incorporated into 6 g Adper Scotchbond 1XT and into 6 g OptiBond FL Prime with the use of a precision balance (Sartorius R180D, Sartorius, Göttingen, Germany) leading to a BAC concentration of 1% [28]. After the adhesive treatment, a split Teflon mold (inner diameter 1.5 mm ≈ bonding area 1.8 mm^2^; height: 2 mm) was clamped to the dentin surface and filled with resin composite (Filtek Z250, 3M ESPE; St. Paul, MN, USA; shade A3, lot number: N605143). The resin composite was covered with a Mylar strip and light-cured for 20 s. All light-curing was performed with an LED-curing unit (Demi, Kerr Corporation, Middleton, WI, USA), and at the beginning and end of each day of specimen preparation the light power density was verified with a radiometer (Demetron L.E.D. Radiometers, Kerr Corporation) to be at least 1000 mW/cm^2^. After light-curing, the SBS specimens were placed in a black photoresistant box in order to avoid any further influence of ambient light. Five minutes after completion of light-curing, the specimens were freed from the Teflon mold. All SBS specimens were then stored in black photoresistant boxes in an incubator (Memmert UM 500, Memmert & Co., Schwabach, Germany) at 37°C and 100% humidity for 24 h. SBS specimens prepared for 1-year storage were transferred to tap water and kept in the incubator at 37°C, with the tap water being changed periodically.

### 2.4. SBS Testing and Failure Mode Determination

After storage, all specimens were subjected to SBS testing by use of a wire (stainless steel, diameter 0.6 mm) at a cross-head speed of 1 mm/min in a universal testing machine (Zwick Z1.0 TN, Zwick GmbH & Co. KG, Ulm, Germany). The maximum force (*F*_max_ [N]) was recorded and the SBS values (MPa) were calculated (*F*_max_ [N]/bonding area [mm^2^]) resulting in 16 SBS values per group for statistical analysis.

After SBS testing, the failure mode of each specimen was determined under a stereomicroscope (Leica ZOOM 2000, Leica, Buffalo, NY, USA) at 40x magnification and classified as (1) cohesive failure in dentin, (2) adhesive failure at dentin/adhesive interface, (3) adhesive failure at adhesive/resin composite interface, (4) cohesive failure in resin composite, or (5) mixed failure (combinations of failure modes 1 to 4).

### 2.5. Statistical Analysis

Due to a lack of normal distribution (Shapiro Wilk's test, *p* = 0.0012), SBS values were analyzed with a nonparametric ANOVA followed by Bonferroni Holm correction for multiple testing. For post hoc analysis, Wilcoxon-Mann-Whitney tests were applied without Bonferroni Holm correction. All calculations were performed with *R* version 3.3.0 (the *R* Foundation for Statistical Computing, Vienna, Austria, https://www.R-project.org) after the significance level had been set at *α* = 0.05. Failure modes after SBS testing were analyzed descriptively.

## 3. Results

**Figure 1:**
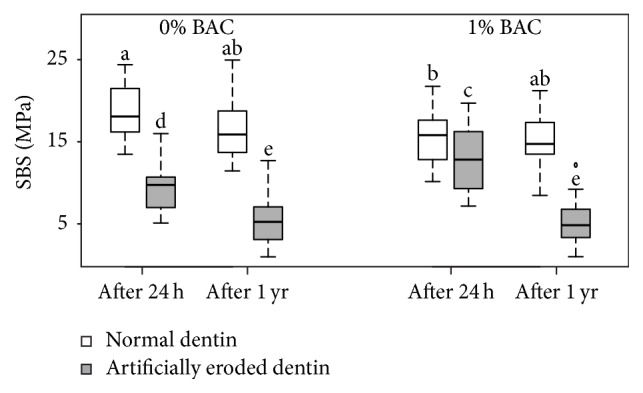
Shear bond strength (SBS (MPa); medians, lower and upper quartiles, and minima and maxima) of Adper Scotchbond 1XT (without/with 1% benzalkonium chloride (BAC) and the four experimental conditions (i.e., normal/artificially eroded dentin and 24 h/1-year (yr) storage); *n* = 16/group). Different lowercase letters show significant differences between the groups.

**Figure 2:**
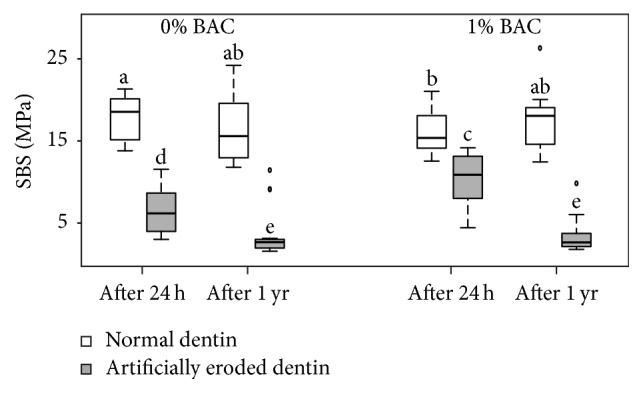
Shear bond strength (SBS (MPa); medians, lower and upper quartiles, and minima and maxima) of OptiBond FL (without/with 1% benzalkonium chloride (BAC) and the four experimental conditions (i.e., normal/artificially eroded dentin and 24 h/1-year (yr) storage); *n* = 16/group). Different lowercase letters show significant differences between the groups.

The SBS values of the two adhesive systems (each without/with 1% BAC incorporation) and the four experimental conditions (i.e., normal/artificially eroded dentin and 24 h/1-year storage) are depicted in Figure 1 for Adper Scotchbond 1XT and in Figure 2 for OptiBond FL. The nonparametric ANOVA showed a significant effect of the factors “type of dentin” (i.e., normal or artificially eroded) and “duration of storage” (i.e., 24 h or 1 year) (both *p* < 0.001) but no significant effect of the factors “BAC incorporation” (*p* = 1.000) and “adhesive system” (*p* = 0.397). The ANOVA also showed significant twofold interactions between the factors “type of dentin”/“BAC incorporation” (*p* = 0.002), “type of dentin”/“duration of storage” (*p* = 0.0003), and “type of dentin”/“adhesive system” (*p* = 0.009) as well as a significant threefold interaction between the factors “type of dentin”/“BAC incorporation”/“duration of storage” (*p* < 0.001). All remaining interactions were not significant (*p* ≥ 0.927). The significant interaction in “type of dentin”/“adhesive system” (*p* = 0.009) implied that the two adhesive systems yielded statistically similar SBS to normal dentin (median SBS values (MPa) without BAC at 24 h/1 year: Adper Scotchbond 1XT 18.1/15.9; OptiBond FL 18.6/15.6) and Adper Scotchbond 1XT yielded higher SBS to eroded dentin than did OptiBond FL (median SBS values (MPa) without BAC at 24 h/1 year: Adper Scotchbond 1XT 9.8/5.3; OptiBond FL 6.2/2.7). However, because of the not statistically significant main effect of adhesive system, the SBS values obtained with Adper Scotchbond 1XT and OptiBond FL were pooled for further post hoc tests. These tests revealed that SBS was significantly higher for normal dentin than for artificially eroded dentin regardless of BAC incorporation and/or duration of storage (*p* < 0.001). On normal dentin, BAC incorporation led to significantly lower SBS after 24 h (*p* = 0.018), but to statistically similar SBS after 1 year (*p* = 1.000). On artificially eroded dentin, BAC incorporation led to significantly higher SBS after 24 h (*p* = 0.001) but to statistically similar SBS after 1 year (*p* = 1.000). Storage for 1 year resulted in a reduction in SBS to artificially eroded dentin (*p* ≤ 0.001), whereas SBS to normal dentin remained stable (*p* ≥ 0.082).

**Figure 3:**
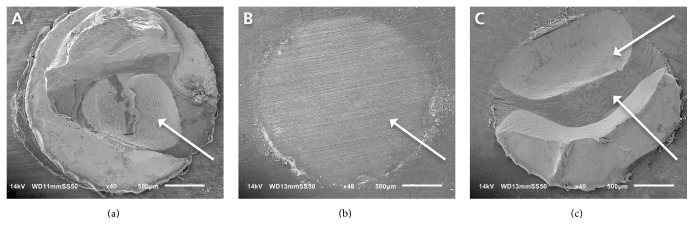
Representative scanning electron micrographs of the three failure modes observed: (a) cohesive failure in dentin, (b) adhesive failure at dentin/adhesive interface, and (c) mixed failure (combination of cohesive failure in dentin and adhesive failure at dentin/adhesive interface with remnants of adhesive system/resin composite).

Of the five afore-listed failure modes, only cohesive failures in dentin (Figure 3(a)), adhesive failures at dentin/adhesive interface (Figure 3(b)), and mixed failures (combinations of cohesive failures in dentin and adhesive failures at dentin/adhesive interface, Figure 3(c)) occurred. The distribution of failure modes after SBS testing is shown in Table 3. For both adhesive systems and both storage durations, the predominant failure mode was adhesive failure at the dentin/adhesive interface with a tendency to even more adhesive failures after 1-year storage.

## 4. Discussion

The present study investigated the effect of benzalkonium chloride (BAC) incorporation into a two-step (Adper Scotchbond 1XT) and a three-step (OptiBond FL) etch-and-rinse adhesive system on the long-term bond strength to normal and to artificially eroded dentin. Since BAC incorporation under certain experimental conditions led to significantly different bond strengths than did the non-BAC-containing control groups, the first null hypothesis was rejected. On normal dentin, BAC incorporation led to a reduction in the 24 h bond strength and a slight increase in the number of adhesive failures at the dentin/adhesive interface, indicating that the incorporation of BAC per se affected the bond-promoting capacity of the two adhesive systems. It may be that the incorporation of BAC upset the optimized monomer-solvent balance in the adhesive, thereby either hampering effective infiltration into and around the collagen fibrils [16] or interfering with the light-induced polymerization to decrease the degree of cure [35]. The result is in contrast to that of Sabatini et al. who even reported a favorable effect on the 24 h microtensile bond strength of adding 1% BAC to the Adper Single Bond Plus adhesive [28]. One explanation for the discrepancy between the two studies is a change in the composition from that of Adper Single Bond Plus to that of Adper Scotchbond 1XT; another is the difference in dentin tubule orientation of the dentin specimens used. Whereas Sabatini et al. [28] performed bonding to occlusal dentin (dentin tubules cut perpendicularly), the present study performed bonding to axial dentin (dentin tubules cut parallel). It has been speculated that occlusal orientation of dentin tubules facilitates migration of the water present in the tubules to the adhesive interface [36]. Sabatini et al. [28] argued that BAC incorporation into the adhesive improves diffusion and infiltration of the adhesive into the water-filled spaces between the collagen fibrils and dentin tubules. Increased exposure to water from the dentin tubules might then explain why incorporation of BAC into the adhesive had a positive effect on occlusal dentin as opposed to axial and less “wet” dentin.

The artificial erosion protocol applied in the present study led to significantly lower bond strengths to dentin and to a tendency to more adhesive failures at the dentin/adhesive interface, regardless of BAC incorporation and/or duration of storage and thus to rejection of the second null hypothesis. This result corroborates the bond strength findings of Zimmerli et al. [29] who, using TEM and the exact same artificial erosion protocol as in the present study, also reported a thicker layer of exposed collagen that could hardly be infiltrated by the adhesives applied. Probable reasons presented for the inefficient hybridization were collapse of the demineralized collagen fibrils and an increased water content that not only prevented the adhesive from fully infiltrating the demineralized zone but also hampered proper polymerization of the adhesive. Thus, it is likely that the lower 24 h bond strength to eroded dentin found in the present study can be attributed to inferior hybridization. The fact that Adper Scotchbond 1XT yielded higher bond strength to eroded dentin than did OptiBond FL seems to reflect a superior capacity of the Adper Scotchbond 1XT adhesive to infiltrate thick demineralization zones and to effectively polymerize in humid conditions. The relatively good performance on eroded dentin is corroborated by studies on its predecessor Adper Single Bond 2 [37, 38].

Whereas BAC incorporation, as mentioned above, caused a reduction in the 24 h bond strength to normal dentin, a positive effect of BAC incorporation on the 24 h bond strength to eroded dentin was found. As previously mentioned, Sabatini et al. [28] speculated that BAC incorporation into the adhesive improves diffusion and infiltration of the adhesive into the water-filled spaces between the collagen fibrils and dentin tubules, an improvement that could be argued to have greater effect on eroded dentin displaying a thicker demineralization zone.

Both etch-and-rinse adhesive systems provided bonds to normal dentin that remained stable over the study period whether or not BAC had been incorporated into the adhesive. As regards the three-step etch-and-rinse adhesive system OptiBond FL, this finding is in agreement with previous studies that, according to the review by de Munck et al., have reported its bonding effectiveness to be unaffected by water storage, thermocycling, and/or mechanical loading [2]. The resistance to bond degradation of OptiBond FL, and other three-step etch-and-rinse adhesive systems, has been attributed to the inclusion of a separate and hydrophobic adhesive resin layer that is presumed to prevent, or at least decrease, the absorption of water within the dentin matrix and thus limit the degradation of collagen by MMPs [39]. Since MMPs are enzymes that require water to hydrolyze the peptide bonds in the collagen molecules [6], it may be that, in the presence of a hydrophobic adhesive resin layer, sealing the acid-etched dentin not only renders the bonded interface more stable but also lets a lower amount of water diffuse through the hybrid layer whereby the collagen hydrolysis by MMPs would be limited [40] and the MMP-inhibiting effect of BAC would not be expressed or would be rendered obsolete. The bond of two-step etch-and-rinse adhesive systems such as Adper Scotchbond 1XT and which do not comprise a separate adhesive layer has generally proved to be less durable, and the positive result found with this adhesive system is thus in conflict with two previous studies [4, 28]. Possible explanations for the fact that Adper Scotchbond 1XT resisted bond degradation are (1) an upgraded formulation as compared to previous versions (e.g., the Adper Single Bond Plus employed by Sabatini et al. [28] that, based on continuing improved understanding on dentin bonding, displayed more complete hybridization) and (2) the test method. Since most degradation processes are diffusion-rate-dependent, the length of the diffusion path is as important a parameter as the diffusion time. Considering that the diameter of the bonded area was 1.5 mm, which is more than the 1 mm width or breadth of most sticks or beams applied in the much used microtensile strength test, it could be argued that water may not have had access to the entire bonding surface, excluding bond degradation at the center of the bond strength specimen. However, the storage time in the present study was much longer than that in several studies, which found a decline in bond strength.

In contrast to the bond strength to normal dentin, the bond strength to eroded dentin deteriorated after 1-year water storage, and the third null hypothesis was therefore partially rejected. The fact that the bond to eroded dentin decreased in consequence of the 1-year water storage corroborates the findings of Zimmerli et al. who reported that bond strength to eroded dentin was more adversely affected by aging [29]. The inferior hybridization mentioned above explains not only the lower immediate bond strength obtained to eroded dentin but also the reduced bond stability caused by enhanced hydrolytic degradation of the imperfect hybrid layer. The lack of ability of BAC incorporation to improve bond stability to eroded dentin may have the following explanation: nonpolymerizable MMP inhibitors, such as BAC and chlorhexidine, bind to dentin electrostatically [41], and noncovalently bound molecules may leach out of the hybrid layer. Leaching of the incorporated BAC would have weakened its MMP-inhibitory effect and minimized or annihilated any positive effect on bond durability. The fact that Sabatini et al. [28] found incorporation of BAC to ensure bond stability may be explained by the 6 months' storage, that is, only half of the 1-year storage in the present study, and in less leaching of the unbound BAC.

The positive result of BAC incorporation on the stability of the bond to normal dentin reported by Sabatini et al. [28] was not confirmed on artificially eroded dentin in the present study. In an effort to gain an increased understanding of the underlying mechanisms for bond durability, future studies should analyze the bonding interfaces on the two dentin substrates and compare adhesives modified with BAC with control adhesives. Considering the discouraging result on eroded dentin, it may be more promising to pursue long-term stability of resin-dentin bonds by (polymerizable) collagen crosslinks [42, 43].

## 5. Conclusions

Based on the results of the present in vitro study, the following conclusions can be drawn:

- Bond strength was significantly lower to artificially eroded dentin than to normal dentin.
- Bond strength to normal dentin did not deteriorate during the 1-year storage.
- Bond strength to artificially eroded dentin deteriorated during the 1-year storage.
- Incorporation of BAC did not increase bond durability.

Consequently, benzalkonium chloride incorporated into the adhesive of a two-step and a three-step adhesive system did not improve bond stability. Other measures to prevent bond degradation and to ensure long-term survival of resin-dentin bonds must be explored.

## Figures and Tables

**Table 1:** Composition of the de- and remineralization solutions used in the artificial erosion protocol.

Solution (at 37°C)	Composition
Demineralization	1% citric acid with pH of 3.5 (anhydrous citric acid; Merck, Darmstadt, Germany)

Remineralization	0.002 g ascorbic acid, 0.58 g NaCl, 0.17 g CaCl_2_, 0.16 g NH_4_Cl, 1.27 g KCl, 0.16 g NaSCN, 0.33 g KH_2_PO_4_, 0.34 g Na_2_HPO_4_ dissolved in 1 L of demineralized water; pH is set to 6.4 with HCl

**Table 2:** Adhesive systems and adhesive treatment (BAC: benzalkonium chloride).

*Adper Scotchbond 1XT* (3M ESPE, St. Paul, MN, USA)	(1) Phosphoric acid etching (Scotchbond Universal Etchant; lot number: 568712; 35% phosphoric acid)	15 s
	Water spray	10 s
	Blot dry (cotton pellet)	—
	(2) Adper Scotchbond 1XT (3 coats; lot number: N597610; BisGMA, HEMA, dimethacrylates, a methacrylate functional copolymer of polyacrylic and polyitaconic acids, ethanol, water, initiators, silane treated silica) (i) without/with 1% BAC	15 s
	Short air dry	(~5 s)
	(3) Light-cure	10 s

*OptiBond FL* (KerrHawe, Scafati, Italy)	(1) Phosphoric acid etching (Kerr Gel Etchant; lot number: 5329366; 37.5% phosphoric acid)	15 s
	Water spray	>15 s
	Air dry	>3 s
	(2) OptiBond Prime (lot number: 5296441; HEMA, GPDM, MMEP, ethanol, water, photoinitiator) (i) without/with 1% BAC	15 s
	Short air dry	(~5 s)
	(3) OptiBond Adhesive (lot number: 5344022; BisGMA, HEMA, GPDM, photoinitiator, filler)	15 s
	Short air dry	(~3 s)
	(4) Light-cure	10 s

**Table 3:** Distribution of failure modes after shear bond strength testing (*n* = 16/group; BAC: benzalkonium chloride).

Adhesive system Dentin	Cohesive failures in dentin (%)	Adhesive failures at dentin/adhesive interface (%)	Mixed failures (%)
*After 24-hour storage*			

Adper Scotchbond 1XT without 1% BAC			
Normal dentin	12.5	62.5	25
Adper Scotchbond 1XT without 1% BAC			
Artificially eroded dentin	0	100	0
Adper Scotchbond 1XT with 1% BAC			
Normal dentin	0	87.5	12.5
Adper Scotchbond 1XT with 1% BAC			
Artificially eroded dentin	0	87.5	12.5
OptiBond FL without 1% BAC			
Normal dentin	6.25	75	18.75
OptiBond FL without 1% BAC			
Artificially eroded dentin	0	100	0
OptiBond FL with 1% BAC			
Normal dentin	6.25	87.5	6.25
OptiBond FL with 1% BAC			
Artificially eroded dentin	0	100	0

*After 1-year storage*			

Adper Scotchbond 1XT without 1% BAC			
Normal dentin	6.25	75	18.75
Adper Scotchbond 1XT without 1% BAC			
Artificially eroded dentin	0	100	0
Adper Scotchbond 1XT with 1% BAC			
Normal dentin	6.25	93.75	0
Adper Scotchbond 1XT with 1% BAC			
Artificially eroded dentin	0	100	0
OptiBond FL without 1% BAC			
Normal dentin	0	81.25	18.75
OptiBond FL without 1% BAC			
Artificially eroded dentin	0	100	0
OptiBond FL with 1% BAC			
Normal dentin	0	93.75	6.25
OptiBond FL with 1% BAC			
Artificially eroded dentin	0	100	0
